# Almond-Derived Peptide Fraction Alleviates DSS-Induced Colitis with Associated Anti-Inflammatory, Antioxidant, and Barrier-Restorative Effects

**DOI:** 10.3390/foods15142419

**Published:** 2026-07-08

**Authors:** Tao Sun, Feiran Xu, Xinmeng Zhang, Zhonghong Wu, Huilian Che, Xiaolong Zhou, Jinfang Liu

**Affiliations:** 1 College of Food Science and Nutritional Engineering, China Agricultural University, Beijing 100083, China; suntao26@xaas.ac.cn (T.S.); feiranxu@cau.edu.cn (F.X.); xinmengz@126.com (X.Z.); chehuilian@cau.edu.cn (H.C.); 2 Xinjiang Academy of Agricultural Sciences, Urumqi 830091, China; wuzhonghong111@163.com; 3 Sichuan Advanced Agricultural and Industrial Institute, China Agricultural University, Chengdu 611430, China

**Keywords:** almond-derived peptides, DSS-induced colitis, epithelial barrier, gut microbiota, transcriptomics, Spearman correlation, host–microbiota interaction

## Abstract

Almond-derived peptide fractions (APs) represent a promising yet underexplored class of plant-based bioactive compounds for ulcerative colitis (UC) management. This study investigated the protective effects of APs against dextran sulfate sodium (DSS)-induced colitis in male BALB/c mice using integrated 16S rRNA sequencing and transcriptomic analysis. Mice received low-dose (200 mg/kg) or high-dose (400 mg/kg) APs during 7-day DSS administration. Both doses significantly attenuated body weight loss, disease activity index, colon shortening, and histopathological damage. Compared with the DSS group, low-dose and high-dose APs reduced disease activity index by 65.9% and 52.4%, increased colon length by 30.0% and 26.5%, decreased histopathological scores by 39.6% and 55.1%, and lowered colonic myeloperoxidase activity by 13.9% and 34.1%, respectively. APs also modulated inflammatory cytokines, improved mucus-barrier-related indices, and enhanced continuous ZO-1 localization at epithelial junctions. Additionally, 16S rRNA sequencing indicated that AP treatment was associated with altered genus-level microbiota profiles after DSS exposure, including changes in Ligilactobacillus, Escherichia-Shigella, and Parabacteroides-related taxa. Transcriptomic analysis suggested dose-associated response patterns: low-dose APs were associated with broader immune-related transcriptional changes, whereas high-dose APs were more closely associated with extracellular matrix organization, focal adhesion, and genes enriched in the PI3K-Akt signaling pathway. These findings support the protective effects of APs in DSS-induced acute colitis and suggest potential associations among microbiota alterations, host transcriptional responses, and mucosal repair.

## 1. Introduction

Ulcerative colitis (UC) is a major subtype of inflammatory bowel disease (IBD), characterized by chronic and relapsing inflammation of the colonic mucosa. Its main clinical manifestations include abdominal pain, diarrhea, and mucopurulent bloody stools [1]. The incidence and prevalence of IBD continue to rise worldwide, making it an important public health burden [2]. The pathogenesis of UC involves complex interactions among genetic susceptibility, environmental factors, gut microbiota dysbiosis, and abnormal mucosal immune responses [3]. Current pharmacological therapies for UC mainly include 5-aminosalicylates, corticosteroids, and immunosuppressants. Although these agents can control symptoms to some extent, long-term use is often associated with adverse effects. Therefore, natural bioactive substances with favorable safety profiles and multiple mechanisms of action have attracted increasing attention as adjunctive or alternative strategies for the prevention and management of IBD.

Food-derived bioactive peptides are small peptides released from proteins through enzymatic hydrolysis, fermentation, or gastrointestinal digestion. They usually contain 2–20 amino acid residues and exhibit various biological activities, including anti-inflammatory, antioxidant, immunomodulatory, and antimicrobial effects [4]. Compared with conventional drugs, food-derived bioactive peptides have received considerable interest because of their relatively low molecular weight, favorable bioavailability, and good safety profile [5]. A systematic review including 11 animal studies showed that food-derived bioactive peptides can protect intestinal health through multiple pathways, such as reducing TNF-α and NF-κB expression, promoting IgA secretion, improving intestinal morphological parameters, and increasing microbial diversity [6]. In recent years, peptides from different food sources, including walnut peptides [7], oyster peptides [8], and wheat peptides [9], have been reported to exert protective effects in DSS-induced colitis models. Their mechanisms involve inhibition of pro-inflammatory signaling pathways, restoration of the intestinal barrier, modulation of gut microbiota composition, and attenuation of oxidative stress. In addition, food proteins and their hydrolytic peptides can indirectly affect host immune responses by regulating gut microbiota composition, forming a “peptide–microbiota–host” interaction network that provides a useful framework for understanding the intestinal protective effects of bioactive peptides [10].

Almond (*Amygdalus communis* L.) is one of the most widely produced tree nuts worldwide. It is rich in protein, approximately 25%, as well as unsaturated fatty acids, vitamins, minerals, and polyphenolic bioactive compounds [11]. Previous studies have linked almond intake with reduced cardiovascular risk, improved lipid profiles, and better glycemic regulation [12]. Almond protein has a balanced essential amino acid composition that conforms to the FAO recommended pattern and is rich in functional amino acid residues such as glutamic acid, arginine, and aspartic acid. These compositional features provide a favorable precursor basis for the release of bioactive peptides after enzymatic hydrolysis. In the field of bioactive peptide research, Udenigwe et al. [13] reported that almond protein hydrolysate fractions suppressed the expression of pro-inflammatory cytokines and cyclooxygenase-2 in lipopolysaccharide-activated macrophages, suggesting the anti-inflammatory potential of almond-derived peptides. Liu et al. [14] further isolated and identified two ACE-inhibitory peptides, Met-His-Thr-Asp-Asp and Gln-His-Thr-Asp-Asp, from almond protein hydrolysates and showed that they protected vascular endothelial function by regulating nitric oxide and endothelin release. These findings indicate that almond-derived peptides may have biological activities beyond anti-inflammatory effects alone. Almond protein hydrolysates have also been confirmed to exhibit antioxidant and angiotensin-converting enzyme inhibitory activities [15]. Taken together, almond protein is a promising substrate for the development of plant-derived functional peptides because of its high-quality amino acid composition and its potential to release diverse bioactive peptides. However, their protective effects in experimental colitis and their associated host–microbiota responses remain unclear. Moreover, whether almond-derived peptides regulate intestinal inflammation through mechanisms comparable to or distinct from other plant-derived peptides has not been investigated in vivo.

The dextran sulfate sodium (DSS)-induced acute colitis model in mice is widely used in UC mechanistic studies and candidate intervention evaluation because its pathological characteristics resemble those of human UC [16]. In the present study, almond-derived peptides were prepared through simulated gastrointestinal digestion, and a DSS-induced acute colitis model was established in BALB/c mice. We systematically evaluated the effects of APs on colitis symptoms, colonic histopathological injury, serum inflammatory cytokines, oxidative stress markers, and intestinal barrier function. In addition, 16S rRNA gene sequencing and transcriptomic analysis were combined to explore potential mechanisms associated with AP-mediated protection from the perspectives of gut microbiota regulation and host gene expression. This study provides experimental evidence for the application of plant-derived bioactive peptides in UC prevention and management and supports the high-value utilization of almond protein resources.

## 2. Materials and Methods

### 2.1. Materials and Reagents

Thirty-two specific-pathogen-free male BALB/c mice, 6 weeks old and weighing 20 ± 2 g, were purchased from Beijing Vital River Laboratory Animal Technology Co., Ltd. (Beijing, China; license number: SYXK [Jing] 2025-0079). Almonds, cultivar Shache No. 1, were provided by the Xinjiang Academy of Agricultural Sciences. Dextran sulfate sodium (DSS; molecular weight 40,000 Da) was purchased from Shanghai Macklin Biochemical Co., Ltd. (Shanghai, China). Paraformaldehyde, 4%, was obtained from BIOSHARP (Hefei, Anhui, China). ELISA kits for mouse tumor necrosis factor-α (TNF-α), mouse TGF-β-induced early gene 1 (TIEG1), mouse interleukin-6 (IL-6), and mouse interleukin-10 (IL-10), as well as assay kits for mouse myeloperoxidase (MPO) and superoxide dismutase (SOD), were supplied by Beijing Dongge Boye Biotechnology Co., Ltd. (Beijing, China). All other chemicals were of analytical grade.

The animal study was reviewed and approved by China Agricultural University Laboratory Animal Welfare and Animal Experimental Ethical Inspection Committee under approval number AW50306202-5-04. All experimental procedures were performed in accordance with institutional guidelines for the care and use of laboratory animals.

### 2.2. Preparation of Almond Protein Isolate and Almond-Derived Peptides

#### 2.2.1. Preparation of Almond Protein Isolate

Almond kernels were shelled, dried at 40 °C for 24 h, mechanically crushed, and passed through a 20-mesh sieve. A 200 g sample was defatted with petroleum ether at a solid-to-liquid ratio of 1:10 (*w*/*v*) under magnetic stirring at 30–60 °C for 1 h. The defatting procedure was repeated once. When the petroleum ether phase became clear, it was discarded, and the residue was air-dried overnight, crushed again, passed through a 60-mesh sieve, packaged, and stored under refrigeration. Defatted almond powder was mixed with deionized water at a ratio of 1:10 (*w*/*v*), and the pH was adjusted to 9.0 with 1 mol/L NaOH. After ultrasonication at 40 °C for 1 h, the pH was readjusted to 9.0. The suspension was allowed to stand for 20 min and then centrifuged at 8000 r/min and 4 °C for 15 min. The supernatant was adjusted to pH 4.5 with 1 mol/L HCl and allowed to stand for 20 min. The supernatant was discarded, and the protein precipitate was collected by centrifugation at 8000 r/min and 4 °C for 15 min. This procedure was repeated once. The precipitate was washed with deionized water until neutral, freeze-dried under vacuum, and stored at −20 °C until use.

#### 2.2.2. Preparation of Almond-Derived Peptides

For simulated gastric digestion, gastric electrolyte solution was prepared by dissolving 3.10 g NaCl, 1.10 g KCl, 0.30 g CaCl_2_·2H_2_O, and 0.60 g NaHCO_3_ in 1 L distilled water. A 1 mol/L sodium acetate solution was prepared and adjusted to pH 5.0. All solutions were preheated to 37 °C. Almond protein, 17.0 g, was mixed with 255 mL gastric electrolyte solution, 59.50 mg pepsin, and 0.51 mL sodium acetate solution in an Erlenmeyer flask. The pH was adjusted to 2–3 with 1 mol/L HCl, and the mixture was incubated at 37 °C and 80 r/min for 2 h. Enzymes were inactivated in a 90 °C water bath for 30 min, followed by centrifugation at 10,000 r/min and 4 °C for 15 min. The supernatant was collected and freeze-dried.

For simulated intestinal digestion, intestinal electrolyte solution was prepared by dissolving 5.40 g NaCl, 0.65 g KCl, and 0.33 g CaCl_2_·2H_2_O in 1 L distilled water. A 7% trypsin-PBS solution and a 4% bile salt-PBS solution were also prepared. To the remaining gastric digest, 50 mL intestinal electrolyte solution, 50 mL trypsin-PBS solution, 100 mL bile salt-PBS solution, 59.50 mg pancreatic lipase, and 13.00 mg trypsin were added. The pH was adjusted to 7.0 with 0.10 mol/L NaOH at 37 °C, and the mixture was incubated at 80 r/min for 2 h. Enzymes were inactivated at 90 °C for 30 min, followed by centrifugation at 10,000 r/min and 4 °C for 15 min. The supernatant was collected and freeze-dried to obtain almond-derived peptides.

### 2.3. DSS-Induced Acute Colitis Model

Six-week-old male BALB/c mice were housed under SPF conditions at 20–24 °C, 40–70% relative humidity, and a 12 h light/dark cycle, with free access to food and water. After 7 days of acclimatization, mice were randomly divided by body weight into four groups (n = 8 per group): normal control group (NC), DSS model group (DSS), low-dose AP group (200 mg/kg, Pep_L), and high-dose AP group (400 mg/kg, Pep_H). The sample size of eight mice per group was selected based on commonly used designs in DSS-induced colitis studies with similar phenotypic and histological endpoints, while also following the reduction principle of animal ethics.

From day 1, all groups except the NC group received 2.5% DSS in drinking water for 7 consecutive days to establish the acute colitis model. Water bottles were cleaned daily, and distilled water or DSS solution was replaced every day. During modeling, mice in the Pep_L and Pep_H groups received 0.2 mL AP solution by oral gavage once daily at the corresponding dose. Mice in the NC and DSS groups received an equal volume of distilled water. Gavage and water replacement were performed daily at 9:00 a.m.

### 2.4. Body Weight and Disease Activity Index Monitoring

During DSS modeling, body weight was recorded daily before gavage. Stool consistency, diarrhea, and rectal bleeding were also monitored. Disease activity index (DAI) was used to quantify disease severity. The scoring criteria are shown in Table 1. DAI was calculated as follows:

(1) DAI = (body weight loss score + stool consistency score + rectal bleeding score)/3

### 2.5. Macroscopic Assessment of Colonic Morphology

On the day after DSS administration ended, mice were fasted for 6 h with free access to water and then sacrificed. Blood was collected from the retro-orbital plexus, allowed to stand, and centrifuged to obtain serum, which was stored at −80 °C. The colon was quickly excised, and adherent fat and fecal contents were removed. The colon was gently opened along the mesenteric border, rinsed with sterile PBS, and placed flat on filter paper. Colon length was measured from the cecum to the distal rectum, and macroscopic morphological changes were recorded.

### 2.6. Histopathological Analysis by Hematoxylin and Eosin Staining

Approximately 1 cm of the middle colon was fixed overnight in 4% paraformaldehyde, dehydrated through graded ethanol, cleared in xylene, embedded in paraffin, and sectioned at approximately 4 μm. Sections were deparaffinized, rehydrated, stained with hematoxylin and eosin (H&E), dehydrated, cleared, and mounted. Colonic mucosal architecture, crypt integrity, and inflammatory cell infiltration were observed under a microscope, and histopathological scoring was performed.

### 2.7. Serum Inflammatory Marker Detection

Blood samples were allowed to stand at room temperature for 4 h and centrifuged at 1000× *g* for 20 min to obtain serum. Serum levels of TNF-α, IL-6, IL-10, and TIEG1 were measured using ELISA kits according to the manufacturers’ instructions. Absorbance was measured at the specified wavelength using a microplate reader, and concentrations were calculated from standard curves. Results are expressed as pg/mL.

### 2.8. Determination of Oxidative Stress Markers in Colon Tissue

Colon tissue samples were accurately weighed, mixed with pre-chilled PBS at an appropriate ratio, homogenized on ice, and centrifuged to obtain supernatants. MPO and SOD activities were measured according to the kit instructions. Concentrations were calculated from standard curves and normalized to tissue weight. Results are expressed as ng/g tissue.

### 2.9. Alcian Blue–Periodic Acid-Schiff Staining

Fixed colon tissues were routinely dehydrated, embedded in paraffin, and sectioned. After deparaffinization and rehydration, sections were stained with Alcian blue (AB) to visualize acidic mucins, followed by periodic acid-Schiff (PAS) staining to detect neutral mucins. Sections were then dehydrated, cleared, and mounted. Goblet cells and mucus secretion were observed under a microscope to assess mucus layer integrity and changes in goblet cell distribution.

### 2.10. Immunohistochemical Staining

Colon tissues were fixed in 4% paraformaldehyde, dehydrated, embedded in paraffin, and sectioned. After deparaffinization and rehydration, antigen retrieval was performed. Endogenous peroxidase activity was blocked with 3% hydrogen peroxide, and nonspecific binding was blocked with blocking solution. Sections were incubated with primary antibody against ZO-1 at 4 °C overnight. The next day, sections were washed, incubated with the corresponding secondary antibody, developed with DAB, counterstained with hematoxylin, dehydrated, cleared, and mounted. ZO-1 expression and distribution at epithelial tight junctions were observed under a microscope.

For each treatment group, 3–5 intact colonic mucosal epithelial regions were randomly selected. ZO-1 junctional continuity was scored at 40× magnification according to the following criteria: 0, almost no specific linear staining; 1, weak positive staining with obvious discontinuity; 2, moderate positive staining with partial continuous linear expression; 3, clear and continuous linear expression at most epithelial junctions. Scoring was independently performed by two observers blinded to group allocation, and the mean score was used for statistical analysis.

### 2.11. Gut Microbiota Analysis by 16S rRNA Gene Sequencing

Cecal contents were collected into sterile cryovials, snap-frozen in liquid nitrogen, and stored at −80 °C. Total genomic DNA was extracted using a soil/fecal genomic DNA extraction kit (Omega Bio-tek, Norcross, GA, USA). DNA quality was assessed by agarose gel electrophoresis and NanoDrop 2000 spectrophotometry (Thermo Fisher Scientific, Waltham, MA, USA). The V3–V4 region of the 16S rRNA gene was amplified using barcoded primers 338F, 5′-ACTCCTACGGGAGGCAGCAG-3′, and 806R, 5′-GGACTACHVGGGTWTCTAAT-3′. Purified and quantified PCR products were sequenced on the Illumina NextSeq 2000 platform using paired-end 300 bp sequencing by Majorbio Bio-Pharm Technology Co., Ltd. (Shanghai, China).

Raw reads were quality-filtered and merged, and DADA2 was used for denoising to obtain amplicon sequence variants (ASVs). Taxonomic annotation was performed using QIIME2 and the Silva 138 database. Analyses included α-diversity indices, β-diversity based on Bray–Curtis distance using principal coordinate analysis (PCoA), community composition at phylum and genus levels, and LEfSe analysis with an LDA threshold > 2.

### 2.12. Transcriptomic Analysis of Colon Tissue

Colon tissues from six mice per group were used for total RNA extraction with TRIzol reagent. RNA integrity was assessed using an Agilent 2100 Bioanalyzer (Agilent Technologies, Santa Clara, CA, USA), and samples with RIN ≥ 7.0 were used for library construction. mRNA was enriched using oligo(dT) magnetic beads, and cDNA libraries were prepared. Sequencing was performed on the Illumina NovaSeq 6000 platform using paired-end 150 bp sequencing by Majorbio Bio-Pharm Technology Co., Ltd. (Shanghai, China).

Raw reads were filtered using fastp and aligned to the mouse reference genome GRCm39 using HISAT2. Gene expression levels were calculated as FPKM values using StringTie. Differential expression analysis was performed using DESeq2, with |log_2_FC| > 1 and padj < 0.05 as the criteria for differentially expressed genes (DEGs). KEGG pathway enrichment analysis was conducted using clusterProfiler with padj < 0.05. DEGs were visualized using volcano plots, heatmaps, and Venn diagrams.

Data Availability Statement: The 16S rRNA gene sequencing data generated in this study have been deposited in the NCBI Sequence Read Archive (SRA) under BioProject accession number PRJNA1472308. The transcriptome sequencing data generated in this study have been deposited in the NCBI SRA under BioProject accession number PRJNA1472542.

### 2.13. Statistical Analysis

Data are presented as mean ± SD. Normality was assessed using the Shapiro–Wilk test, and homogeneity of variance was assessed using Levene’s test. For normally distributed data with homogeneous variance, one-way ANOVA followed by Tukey’s post hoc test was used for multiple comparisons. For non-normally distributed data, the Kruskal–Wallis test was used. Body weight and DAI were recorded longitudinally; however, endpoint comparisons were used as the primary statistical analysis because the main objective was to evaluate group differences after DSS exposure. For microbiota α-diversity, the Kruskal–Wallis test was applied. β-diversity was assessed by PCoA based on Bray–Curtis distance, and group separation was tested by ANOSIM. LEfSe analysis was performed with an LDA threshold > 2.0. For transcriptomic data, differential expression analysis was performed using DESeq2, with |log_2_FC| > 1 and adjusted *p* < 0.05 as the screening criteria. The Benjamini–Hochberg method was used for false discovery rate correction. Correlation analysis among representative microbial taxa, transcriptomic markers, and colitis-related phenotypic indicators was performed using Spearman’s rank correlation analysis, and the results were visualized as a correlation heatmap. Significance levels were indicated as * *p* < 0.05, ** *p* < 0.01, and *** *p* < 0.001. A value of *p* < 0.05 was considered statistically significant.

## 3. Results

### 3.1. Almond-Derived Peptides Attenuated DSS-Induced Clinical Symptoms and Macroscopic Colonic Injury

Body weight loss, increased DAI, and colon shortening are common phenotypic changes after DSS-induced acute colitis and directly reflect successful model establishment and the protective effect of interventions [17]. Colon shortening is mainly caused by tissue remodeling resulting from intestinal wall edema, muscular layer thickening, and extensive inflammatory cell infiltration, and is an important macroscopic indicator of colitis severity and treatment efficacy [18]. Food-derived bioactive peptides have been shown to suppress body weight loss, reduce DAI scores, and alleviate colon shortening in DSS-induced colitis models, with mechanisms involving anti-inflammatory, antioxidant, and barrier-repair effects [19,20].

**Figure 1:**
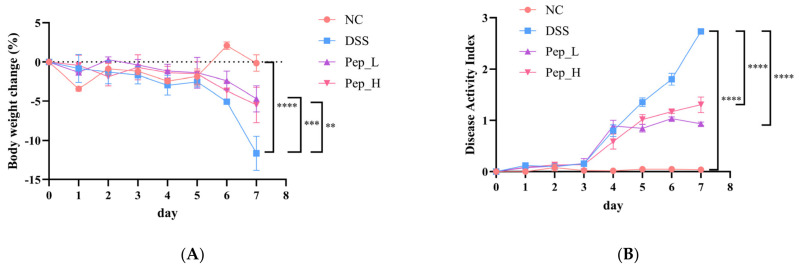

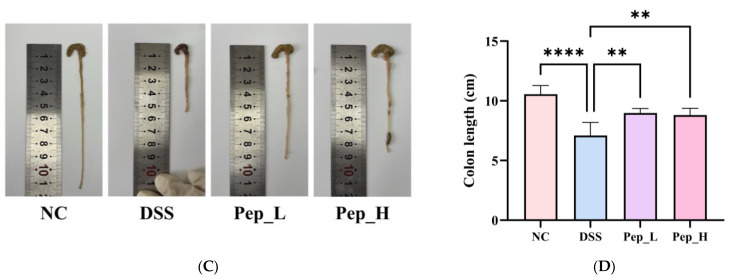
Almond-derived peptides alleviate DSS-induced clinical symptoms and colon shortening in mice. (**A**) Body weight change rate. (**B**) Disease activity index (DAI). (**C**) Representative colon images from each group. (**D**) Colon length. NC, normal control group; DSS, dextran sulfate sodium -induced model group; Pep_L, low-dose almond-derived peptide group; Pep_H, high-dose almond-derived peptide group. Data are presented as mean ± SD. ** *p* < 0.01; *** *p* < 0.001; **** *p* < 0.0001. n = 8 per group.

As shown in Figure 1A,B, body weight in the NC group remained generally stable, and DAI scores stayed low throughout the experiment. In contrast, mice in the DSS group showed continuous body weight loss and increased DAI scores, indicating a marked disease burden caused by DSS treatment. Relative to the DSS group, both the Pep_L and Pep_H groups alleviated body weight loss and reduced DAI scores to varying degrees. On the final day of intervention, DAI scores were reduced by 65.9% in the Pep_L group and 52.4% in the Pep_H group relative to the DSS group.

Macroscopic colon images (Figure 1C,D) showed that the NC group had intact colon morphology and normal colon length. The DSS group showed obvious colon shortening, accompanied by wall thickening, hyperemia, and edema. Both Pep_L and Pep_H groups showed clear improvement compared with the DSS group, and the colonic appearance of the Pep_L group was closer to that of the NC group. AP treatment significantly increased colon length by 30.0% in the Pep_L group and 26.5% in the Pep_H group compared with the DSS group (Pep_L vs. DSS, *p* = 0.0027; Pep_H vs. DSS, *p* = 0.0061).

### 3.2. Almond-Derived Peptides Alleviated DSS-Induced Colonic Histopathological Injury

H&E staining is a routine method for evaluating histological injury in colitis. After DSS treatment, the colon typically shows crypt destruction, epithelial shedding, submucosal edema, and inflammatory cell infiltration, and these changes are closely related to disease severity.

**Figure 2:**
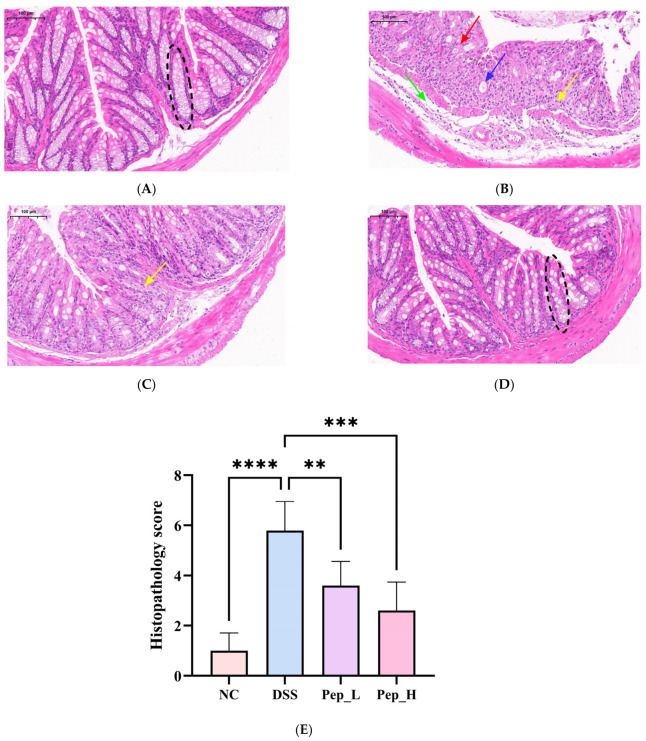
Almond-derived peptides attenuate DSS-induced histopathological damage in mouse colon tissue. Representative hematoxylin and eosin (H&E)-stained colon sections from the (**A**) NC group, (**B**) DSS group, (**C**) Pep_L group, and (**D**) Pep_H group. (**E**) Histopathological score. Black dashed ellipses indicate normal crypt architecture; red arrows indicate ulceration; yellow arrows indicate inflammatory cell infiltration; blue arrows indicate crypt dilation surrounding ulcers; green arrows indicate submucosal edema. Group abbreviations are defined in Figure 1. Data are presented as mean ± SD. ** *p* < 0.01; *** *p* < 0.001; **** *p* < 0.0001. n = 8 per group. Scale bars = 100 μm.

As shown in Figure 2A–D, the NC group exhibited intact colonic mucosal architecture, regularly arranged crypts, and little inflammatory cell infiltration. In the DSS group, large areas of ulceration were observed, accompanied by considerable infiltration of granulocytes, lymphocytes, and other inflammatory cells. The epithelium was flattened, glandular lumina were enlarged, and the submucosa showed edema with loosely arranged connective tissue. Compared with the DSS group, the Pep_L group showed reduced pathological injury, although some crypt disorganization and inflammatory infiltration remained. The Pep_H group showed more evident improvement, with relatively intact crypt structure and reduced inflammatory infiltration.

Histopathological scores were consistent with the H&E observations (Figure 2E). The DSS group had the highest score, whereas AP intervention significantly reduced the scores by 39.6% in the Pep_L group and 55.1% in the Pep_H group (Pep_L vs. DSS, *p* = 0.0087; Pep_H vs. DSS, *p* = 0.0003). These findings, together with the changes in body weight, DAI, and colon length, indicate that APs improved not only the disease phenotype but also colonic mucosal injury.

### 3.3. Almond-Derived Peptides Regulated DSS-Induced Imbalance of Serum Inflammatory Cytokines

Cytokine imbalance is a key event in the development and progression of DSS-induced colitis. TNF-α and IL-6 promote immune cell recruitment, epithelial cell injury, and inflammatory cascade amplification, and they are commonly used indicators of the pro-inflammatory state in colitis [1]. IL-10 is an important anti-inflammatory cytokine that maintains intestinal immune tolerance; IL-10-deficient mice spontaneously develop chronic enterocolitis [21].

**Figure 3:**
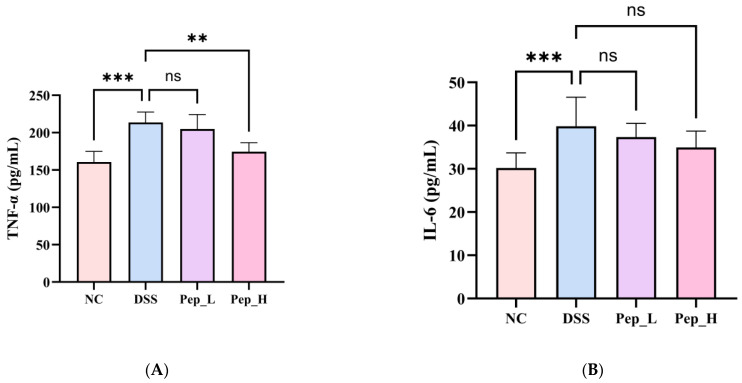

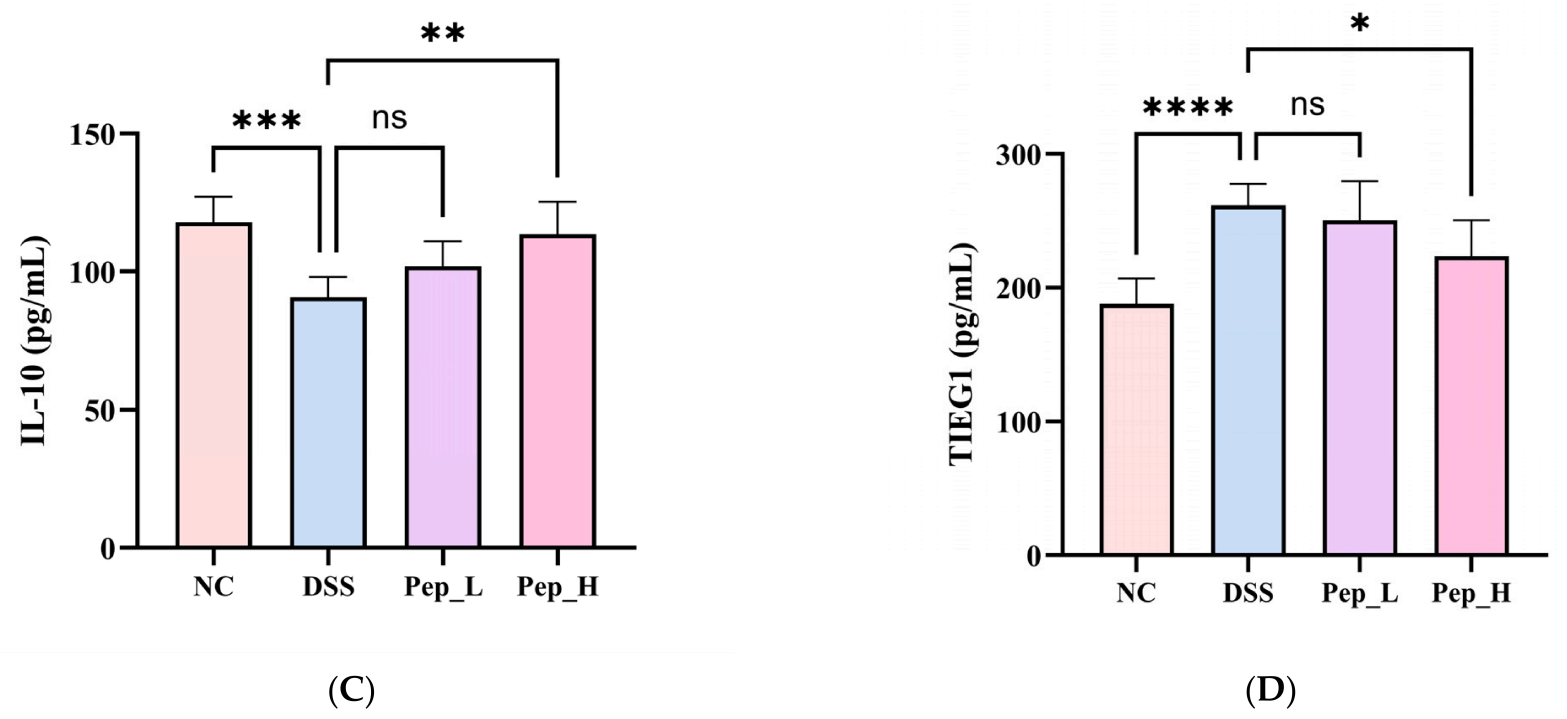
Almond-derived peptides modulate serum inflammatory cytokines in DSS-induced colitis mice. (**A**) Serum tumor necrosis factor-α (TNF-α) levels. (**B**) Interleukin-6 (IL-6) levels. (**C**) Interleukin-10 (IL-10) levels. (**D**) TGF-β-induced early gene 1 (TIEG1) levels. Group abbreviations are defined in Figure 1. Data are presented as mean ± SD. ns, not significant; * *p* < 0.05; ** *p* < 0.01; *** *p* < 0.001; **** *p* < 0.0001. n = 8 per group.

Serum cytokine analysis (Figure 3A–C) showed that TNF-α and IL-6 levels were markedly increased in the DSS group, whereas IL-10 levels were decreased, indicating a pronounced pro-inflammatory state after DSS induction. After AP intervention, TNF-α and IL-6 levels decreased, while IL-10 levels increased, with a more obvious regulatory effect in the Pep_H group. These results were consistent with the reduced inflammatory cell infiltration observed in H&E staining, suggesting that APs improved the DSS-induced pro-inflammatory/anti-inflammatory imbalance.

It should be noted that the change in TIEG1 differed from that of IL-10. As shown in Figure 3D, TIEG1 levels were highest in the DSS group and lowest in the NC group, while the Pep_L and Pep_H groups showed intermediate levels. TIEG1, also known as KLF10, is a TGF-β-induced early response gene involved in the regulation of TGF-β/Smad signaling. Under inflammatory injury conditions, TGF-β-related signaling may be activated as part of tissue repair and immune regulatory responses. Therefore, the increase in TIEG1 in the DSS group more likely reflects a compensatory response under severe inflammatory stress [22,23]. The reduction of TIEG1 after AP treatment does not necessarily indicate suppression of normal repair; rather, it is consistent with decreased compensatory activation after the inflammatory burden was reduced. The reported inhibition of TNF-α and other pro-inflammatory mediators by almond protein hydrolysates in macrophages also supports this interpretation [13].

### 3.4. Almond-Derived Peptides Alleviated DSS-Induced Oxidative Stress

Oxidative stress and inflammation reinforce each other and represent an important mechanism of tissue injury in DSS-induced colitis. Infiltrating inflammatory cells produce large amounts of reactive oxygen species, which further damage lipids, proteins, and DNA and aggravate barrier disruption. MPO is mainly derived from neutrophils, and its activity is commonly used to reflect neutrophil infiltration in intestinal tissue. SOD is a key antioxidant enzyme that removes superoxide anions and reflects the antioxidant defense capacity of the body.

**Figure 4:**
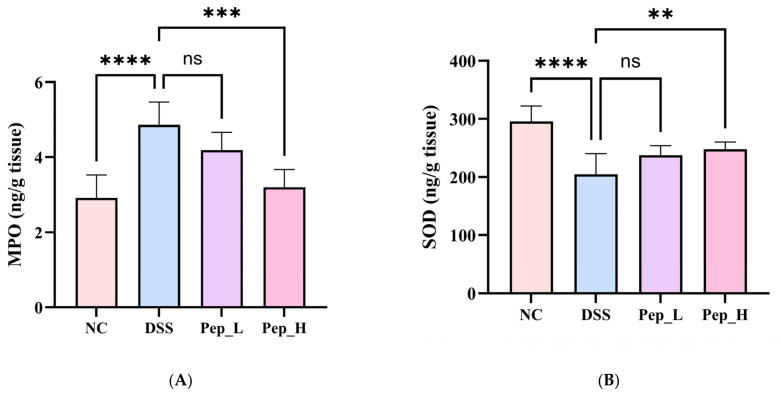
Almond-derived peptides alleviate oxidative stress in the colon tissue of DSS-induced colitis mice. (**A**) Myeloperoxidase (MPO) activity in colon tissue. (**B**) Superoxide dismutase (SOD) activity in colon tissue. Group abbreviations are defined in Figure 1. Data are presented as mean ± SD. ns, not significant; ** *p* < 0.01; *** *p* < 0.001; **** *p* < 0.0001. n = 8 per group.

As shown in Figure 4A,B, MPO activity in colon tissue was increased in the DSS group, whereas SOD activity was decreased, indicating clear inflammatory infiltration and oxidative stress after DSS induction. Intervention with APs reduced colonic MPO activity by 13.9% in the Pep_L group and 34.1% in the Pep_H group and elevated SOD activity, with the Pep_H group exhibiting superior effects. These results were consistent with the reduced inflammatory infiltration in H&E staining and the decrease in serum pro-inflammatory cytokines.

Food-derived peptides often contain hydrophobic amino acids, aromatic amino acids, or sulfur-containing amino acid residues, which may contribute to free radical scavenging, metal ion chelation, and regulation of antioxidant signaling pathways [24]. Wheat peptides and oat peptides have been reported to improve DSS-induced oxidative injury and intestinal barrier disruption by regulating the Keap1-Nrf2 axis [9,25].

### 3.5. Almond-Derived Peptides Improved DSS-Induced Goblet Cell Loss and Mucus Barrier Disruption

MUC2 secreted by goblet cells is a key component of the colonic mucus barrier and plays an important role in separating the epithelium from the luminal microenvironment. MUC2 deficiency can lead to spontaneous colitis [26]. AB-PAS staining simultaneously visualizes acidic and neutral mucins and is commonly used to observe goblet cells and mucus secretion.

**Figure 5:**
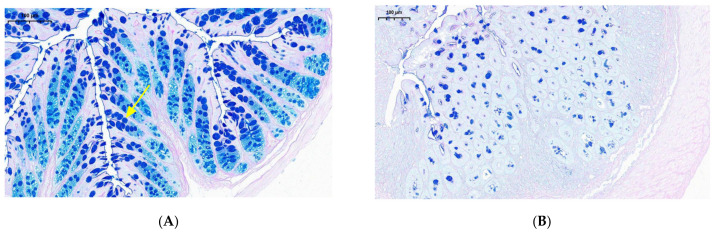

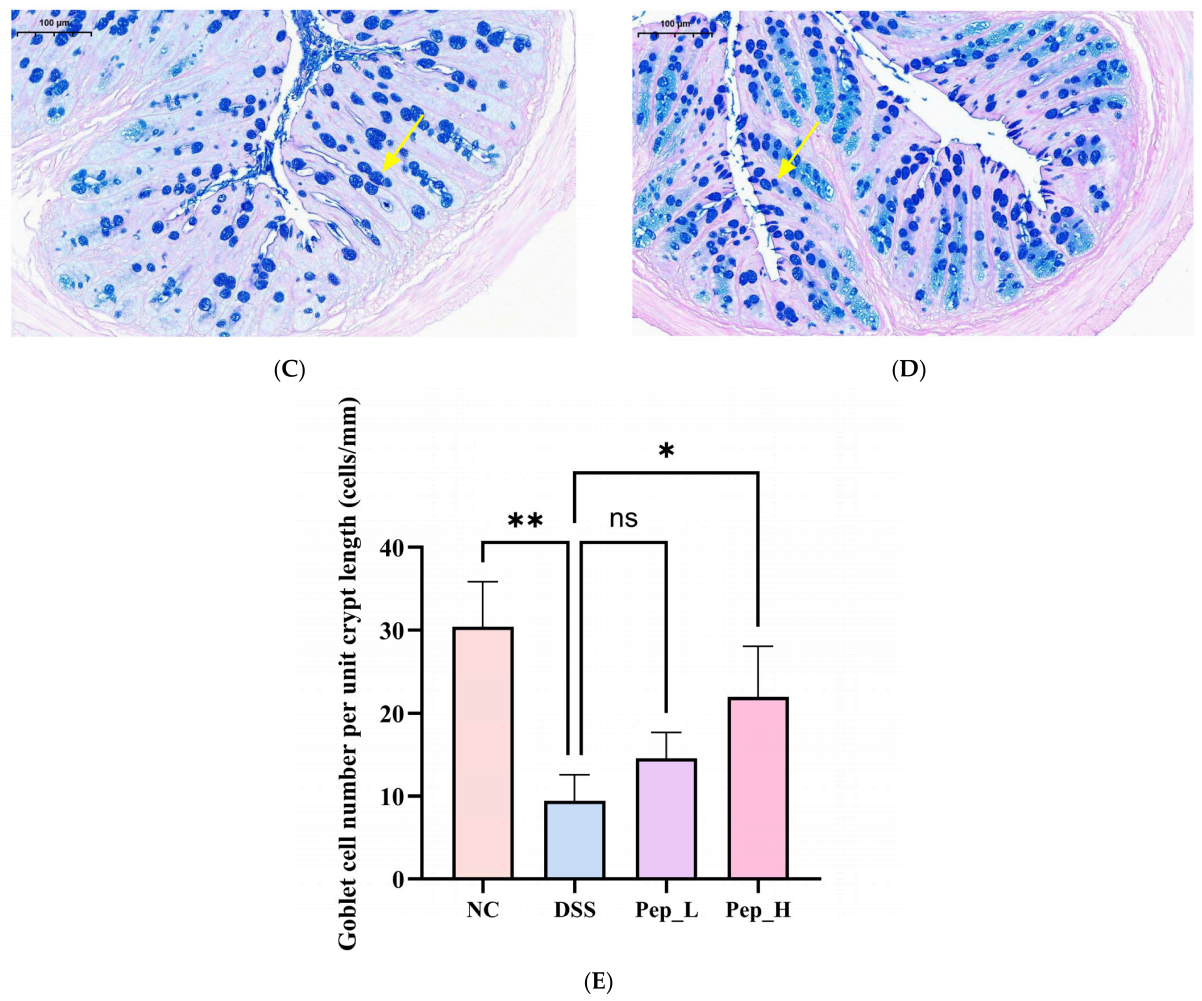
Almond-derived peptides improve the colonic mucus barrier in DSS-induced colitis mice. Representative Alcian blue–periodic acid-Schiff (AB-PAS)-stained colon sections from the (**A**) NC group, (**B**) DSS group, (**C**) Pep_L group, and (**D**) Pep_H group. (**E**) Goblet cell number per unit crypt length. Yellow arrows indicate goblet cells. Group abbreviations are defined in Figure 1. Data are presented as mean ± SD. ns, not significant; * *p* < 0.05; ** *p* < 0.01. n = 8 per group. Scale bars = 100 μm.

AB-PAS staining (Figure 5A–D) showed abundant goblet cells in the colonic crypts of the NC group, with a relatively uniform distribution. In the DSS group, goblet cells were markedly reduced, and the positively stained area was decreased, indicating impaired mucus secretion. The Pep_L group showed a non-significant increasing trend in goblet cell number, whereas the Pep_H group significantly increased goblet cell number compared with the DSS group, accompanied by intact and continuously distributed positive cells in crypts (Figure 5E, Pep_L vs. DSS, *p* = 0.4271; Pep_H vs. DSS, *p* = 0.0279).

### 3.6. Almond-Derived Peptides Enhanced Continuous Linear ZO-1 Expression at Colonic Epithelial Junctions

ZO-1 is an important scaffolding protein in the tight junction complex and plays a central role in maintaining intercellular junctions and paracellular permeability. DSS treatment often causes disrupted ZO-1 localization and reduced expression, accompanied by tight junction damage, which increases intestinal permeability and aggravates inflammation [27]. To avoid the influence of nonspecific background staining in severely damaged tissue areas on positive-area analysis, ZO-1 junctional continuity was further scored under high magnification.

**Figure 6:**
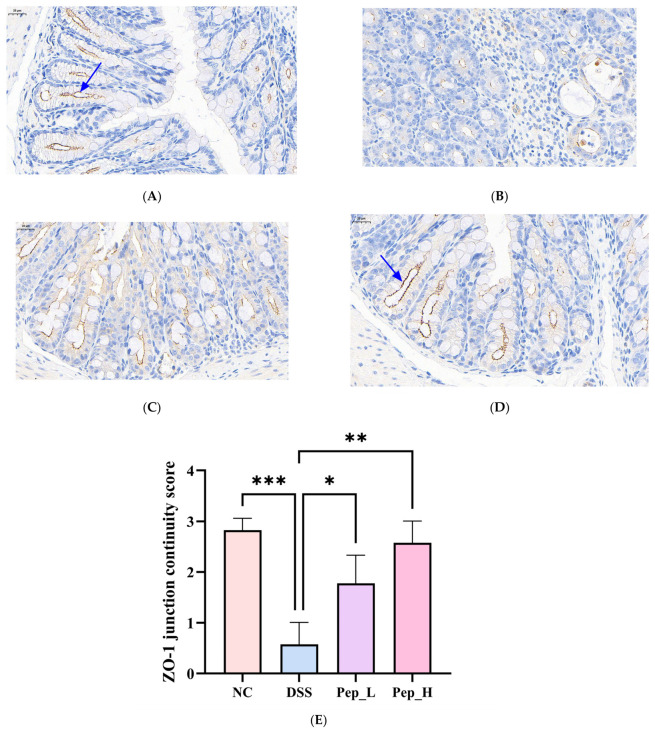
Almond-derived peptides enhance continuous linear ZO-1 expression at colonic epithelial tight junctions. Representative immunohistochemical staining of ZO-1 in colon sections from the (**A**) NC group, (**B**) DSS group, (**C**) Pep_L group, and (**D**) Pep_H group. (**E**) ZO-1 junctional continuity score. Blue arrows indicate continuous linear ZO-1 expression at epithelial junctions. Group abbreviations are defined in Figure 1. Data are presented as mean ± SD. * *p* < 0.05; ** *p* < 0.01; *** *p* < 0.001. n = 8 per group. Scale bars = 25 μm.

As shown in Figure 6A–D, ZO-1 in the NC group was mainly distributed at colonic epithelial junctions in a continuous linear pattern. In the DSS group, ZO-1 junctional localization was clearly impaired, with fewer linear structures, discontinuous staining, and disorganized distribution. After AP intervention, the structural localization of ZO-1 at epithelial junctions was restored, with more pronounced improvement in the Pep_H group. The continuity score (Figure 6E) was significantly lower in the DSS group than in the NC group, while both Pep_L and Pep_H groups showed higher scores than the DSS group. These results indicate that APs reduced DSS-induced disruption of ZO-1 junctional continuity (Pep_L vs. DSS, *p* = 0.0389; Pep_H vs. DSS, *p* = 0.0017).

### 3.7. Almond-Derived Peptides Regulated DSS-Induced Changes in Gut Microbiota Structure

#### 3.7.1. Alpha Diversity Analysis Showed That DSS Disturbed Gut Microbial Diversity

**Figure 7:**
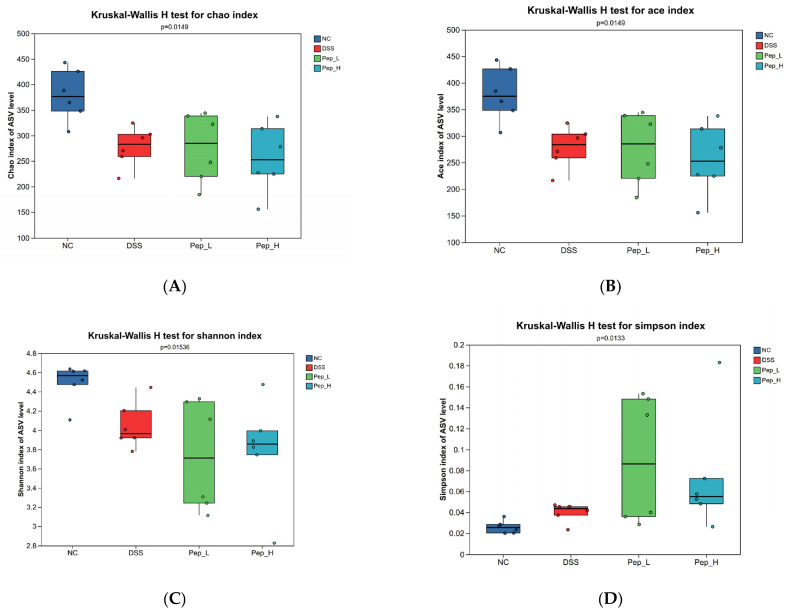
Effects of almond-derived peptides on α-diversity indices of gut microbiota in DSS-induced colitis mice. (**A**) Chao index. (**B**) ACE index. (**C**) Shannon index. (**D**) Simpson index. Group abbreviations are defined in Figure 1. Statistical significance was determined using the Kruskal–Wallis test. *p* < 0.05 indicates a statistically significant difference. n = 6 per group.

To evaluate the effects of different treatments on gut microbial richness and evenness, α-diversity was analyzed using the Chao, ACE, Shannon, and Simpson indices. As shown in Figure 7A–D, the Kruskal–Wallis test indicated significant overall differences among the four groups for the Chao, ACE, Shannon, and Simpson indices, with *p* values of 0.0149, 0.0149, 0.01536, and 0.0133, respectively.

Compared with the NC group, the DSS group showed decreased Chao and ACE indices, suggesting that DSS treatment reduced gut microbial richness. At the same time, the Shannon index decreased and the Simpson index increased, indicating that DSS disrupted community evenness and overall diversity. These findings are consistent with the typical dysbiosis observed in DSS-induced colitis models [16,28].

After AP intervention, the Chao and ACE indices in the Pep_L group increased compared with those in the DSS group, suggesting that low-dose APs partially improved microbial richness. In contrast, the Pep_H group did not show obvious recovery in richness or evenness indices. Overall, α-diversity in both AP-treated groups did not return to the level of the NC group, indicating that the regulatory effects of APs on DSS-induced dysbiosis cannot be evaluated simply by diversity values. Previous studies have shown that improvement of host phenotype during colitis remission or intervention is not necessarily accompanied by full recovery of overall microbial diversity; changes in specific microbial taxa and functional characteristics may have more direct biological relevance [29,30]. Therefore, β-diversity, genus-level composition, and differential taxa analyses were further conducted to evaluate the effects of APs on gut microbiota structure.

#### 3.7.2. Beta Diversity Analysis Indicated Microbiota Restructuring After AP Intervention

**Figure 8:**
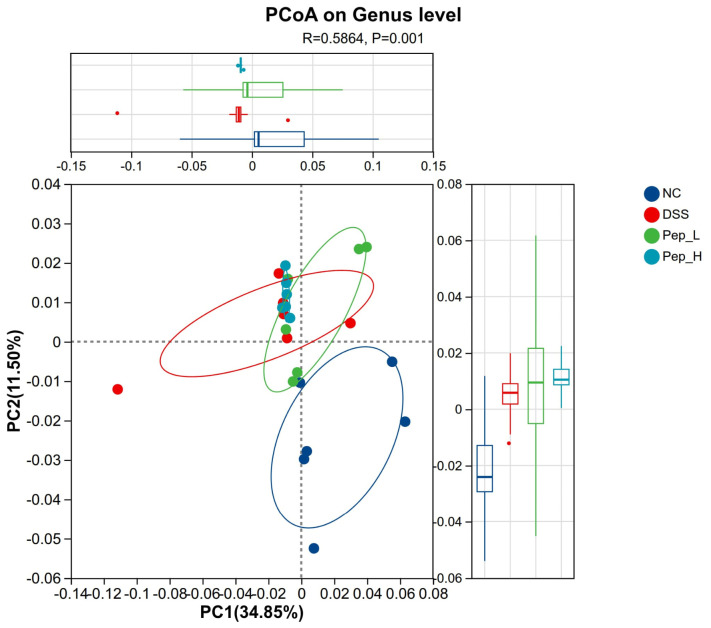
Principal coordinate analysis of gut microbiota structure based on Bray–Curtis dissimilarity. Principal coordinate analysis (PCoA) was performed at the genus level based on Bray–Curtis dissimilarity. Each point represents an individual sample. Group abbreviations are defined in Figure 1. Group differences were assessed by analysis of similarities (ANOSIM; *R* = 0.5864, *p* = 0.001). n = 6 per group.

PCoA analysis at the genus level showed clear differences in gut microbiota structure among groups (Figure 8). ANOSIM analysis yielded R = 0.5864 and *p* = 0.001. PC1 and PC2 explained 34.85% and 11.50% of community variation, respectively.

Samples from the NC group were mainly distributed in the negative region of PC2, whereas samples from the DSS group were clearly separated from those of the NC group, indicating that DSS treatment markedly altered the overall gut microbiota structure. After AP intervention, the distributions of the Pep_L and Pep_H groups differed from that of the NC group and shifted away from the DSS group to some extent, although partial overlap remained among treatment groups. The Pep_L group showed a wider distribution, suggesting individual variation in microbial responses under low-dose treatment. The Pep_H group showed relatively concentrated clustering and was mainly distributed in the positive region of PC2, indicating a more consistent change in microbiota structure after high-dose treatment. Overall, AP intervention did not simply restore the microbiota to the NC-like state; rather, it appeared to induce community restructuring after DSS disturbance.

#### 3.7.3. Genus-Level Composition and LEfSe Analysis Revealed Group-Specific Taxonomic Profiles

**Figure 9:**
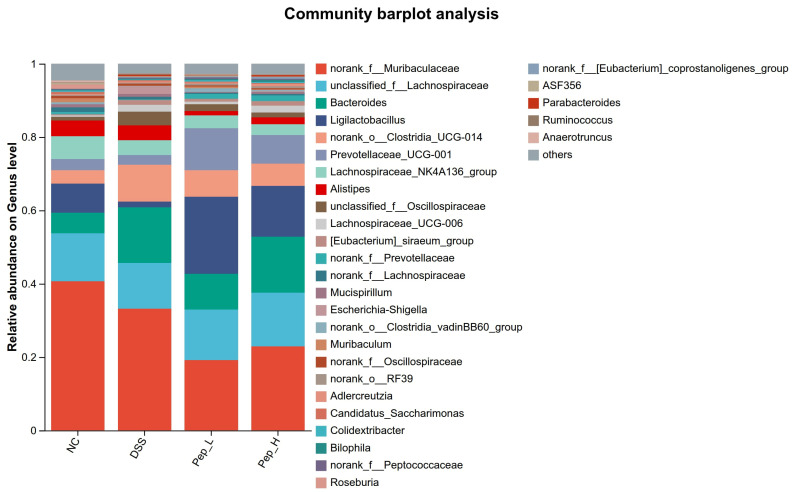
Genus-level taxonomic composition of gut microbiota in different treatment groups. The stacked bar plot shows the relative abundance of dominant bacterial genera and unclassified genus-level taxonomic groups in the NC, DSS, Pep_L, and Pep_H groups. Group abbreviations are defined in Figure 1. n = 6 per group.

Genus-level community composition further revealed specific changes in microbiota structure among groups. The community bar plot (Figure 9) showed that the NC group had a high relative abundance of norank_f__Muribaculaceae, together with unclassified_f__Lachnospiraceae, *Bacteroides*, *Ligilactobacillus*, Lachnospiraceae_NK4A136_group, and *Alistipes*. After DSS treatment, the relative abundance of norank_f__Muribaculaceae decreased, while the proportions of *Bacteroides*, *Muribaculum*, *Escherichia-Shigella*, and some Lachnospiraceae-related taxa changed, indicating a marked shift in dominant bacterial composition after DSS induction.

After AP intervention, the microbiota composition differed between dose groups. In the Pep_L group, the relative abundance of norank_f__Muribaculaceae decreased further, whereas *Ligilactobacillus* and Prevotellaceae_UCG-001 increased. In the Pep_H group, *Ligilactobacillus* remained relatively abundant, while *Bacteroides* and unclassified_f__Lachnospiraceae increased compared with the Pep_L group. These results suggest that both low-dose and high-dose APs altered the microbiota composition after DSS exposure, although the direction of dominant taxa remodeling was not identical.

**Figure 10:**
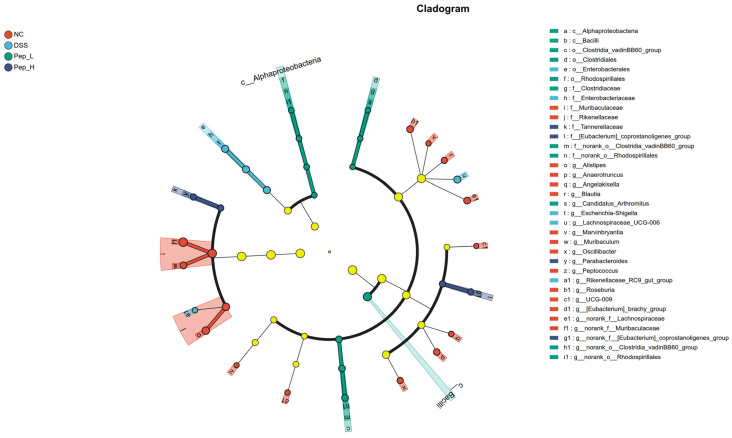
LEfSe cladogram showing the effects of almond-derived peptides on gut microbiota composition in DSS-induced colitis mice. Differentially abundant taxa among the NC, DSS, Pep_L, and Pep_H groups were identified using linear discriminant analysis effect size (LEfSe). Group abbreviations are defined in Figure 1. n = 6 per group.

LEfSe analysis from phylum to genus level (Figure 10) showed that the NC group was mainly enriched in f__Muribaculaceae, g__norank_f__Muribaculaceae, f__Rikenellaceae, g__Alistipes, g__Roseburia, g__Muribaculum, g__Marvinbryantia, g__Peptococcus, and g__Anaerotruncus. The DSS group was mainly enriched in f__Enterobacteriaceae, o__Enterobacterales, g__Escherichia-Shigella, g__Lachnospiraceae_UCG-006, and g__Rikenellaceae_RC9_gut_group. Enterobacteriaceae and *Escherichia-Shigella* have been reported to expand under intestinal inflammatory conditions and are often associated with mucosal barrier injury, enhanced oxidative stress, and inflammatory responses [16,31].

The two AP doses showed different characteristic taxa. The Pep_L group was mainly enriched in c__Bacilli, o__Clostridia_vadinBB60_group, g__norank_o__Clostridia_vadinBB60_group, o__Clostridiales, f__Clostridiaceae, and g__Candidatus_Arthromitus. The Pep_H group was mainly enriched in g__Parabacteroides, f__Tannerellaceae, and [Eubacterium]_coprostanoligenes_group-related taxa. The community bar plot also showed that the relative abundance of *Ligilactobacillus* increased in both Pep_L and Pep_H groups compared with the DSS group, suggesting that lactic acid bacteria-related taxa were among the microbial features altered after AP intervention [32]. Previous studies have shown that lactic acid bacteria can alleviate DSS-induced colitis by regulating gut microbiota composition and inhibiting inflammatory pathways such as TLR4/MyD88/NF-κB [33].

LEfSe analysis identified *Parabacteroides-* and Tannerellaceae-related taxa as characteristic taxa in the Pep_H group in the multi-group comparison. However, because the relative abundance of *Parabacteroides* was comparable between the DSS and Pep_H groups, this result should not be interpreted as AP-induced enrichment of Parabacteroides. Previous studies have shown that *Parabacteroides distasonis* can regulate intestinal immunity and alleviate experimental colitis [34], and may act synergistically with *Akkermansia muciniphila* to promote ILC3-related mucosal protective responses [35]. Nevertheless, because species-level identification and functional validation were not performed in the present study, these findings should be regarded only as biological context rather than evidence for a specific role of *Parabacteroides* in AP-mediated protection.

Taken together, α-diversity, PCoA, community composition, and LEfSe analyses indicate that the microbiota after AP intervention was not simply restored to the NC-like structure but was more likely reorganized after DSS disturbance. The two AP doses showed different effects on microbiota structure. The low-dose group showed partial recovery in richness indices, whereas the high-dose group showed a distinct microbial profile based on LEfSe analysis. Previous studies have suggested that microbial communities do not necessarily return completely to their initial state after disturbance but may form alternative stable states with different compositions and relatively stable functions [36]. In this study, AP intervention was associated with different microbiota response patterns between the two dose groups, resulting in community states distinct from that of the NC group.

### 3.8. Almond-Derived Peptides Were Associated with Changes in DSS-Induced Transcriptional Abnormalities in Colon Tissue

#### 3.8.1. Differential Gene Expression Analysis: Dose-Associated Transcriptional Response Patterns

**Figure 11:**
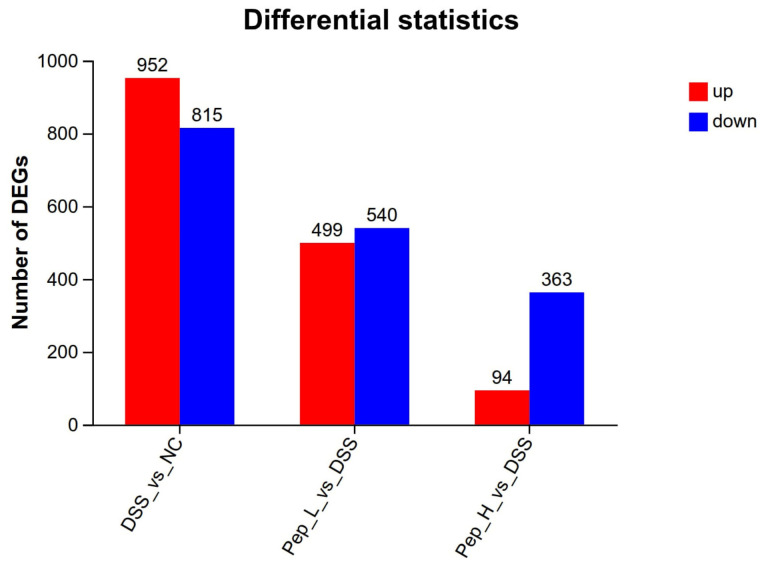
Statistical summary of differentially expressed genes among treatment comparisons. The numbers of upregulated and downregulated differentially expressed genes (DEGs) are shown for the DSS vs. NC, Pep_L vs. DSS, and Pep_H vs. DSS comparisons. Group abbreviations are defined in Figure 1. n = 6 per group.

To further analyze the effects of APs on DSS-induced transcriptional changes in colon tissue, differentially expressed genes (DEGs) were analyzed among groups. As shown in Figure 11, 1767 DEGs were identified in the DSS_vs_NC comparison, including 952 upregulated and 815 downregulated genes, indicating that DSS treatment caused extensive transcriptional changes in colon tissue. In the Pep_L_vs_DSS comparison, 1039 DEGs were identified, including 499 upregulated and 540 downregulated genes. In the Pep_H_vs_DSS comparison, 457 DEGs were identified, including 94 upregulated and 363 downregulated genes. Compared with the Pep_H group, the Pep_L group showed a larger number of DEGs, indicating a broader transcriptional response pattern in the low-dose group. The Pep_H group had fewer DEGs and was mainly characterized by downregulated genes, indicating a more restricted DEG profile in the high-dose group.

**Figure 12:**
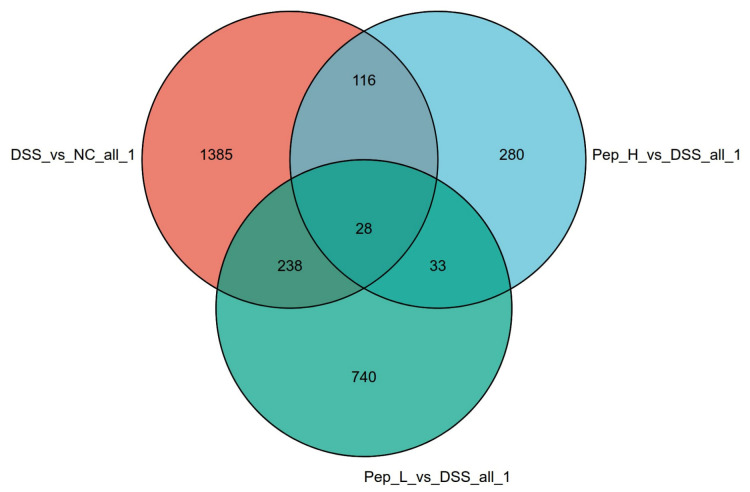
Venn diagram of differentially expressed genes among treatment comparisons. The Venn diagram shows the overlap and unique distribution of differentially expressed genes among the DSS vs. NC, Pep_L vs. DSS, and Pep_H vs. DSS comparisons. Group abbreviations are defined in Figure 1. n = 6 per group.

**Figure 13:**
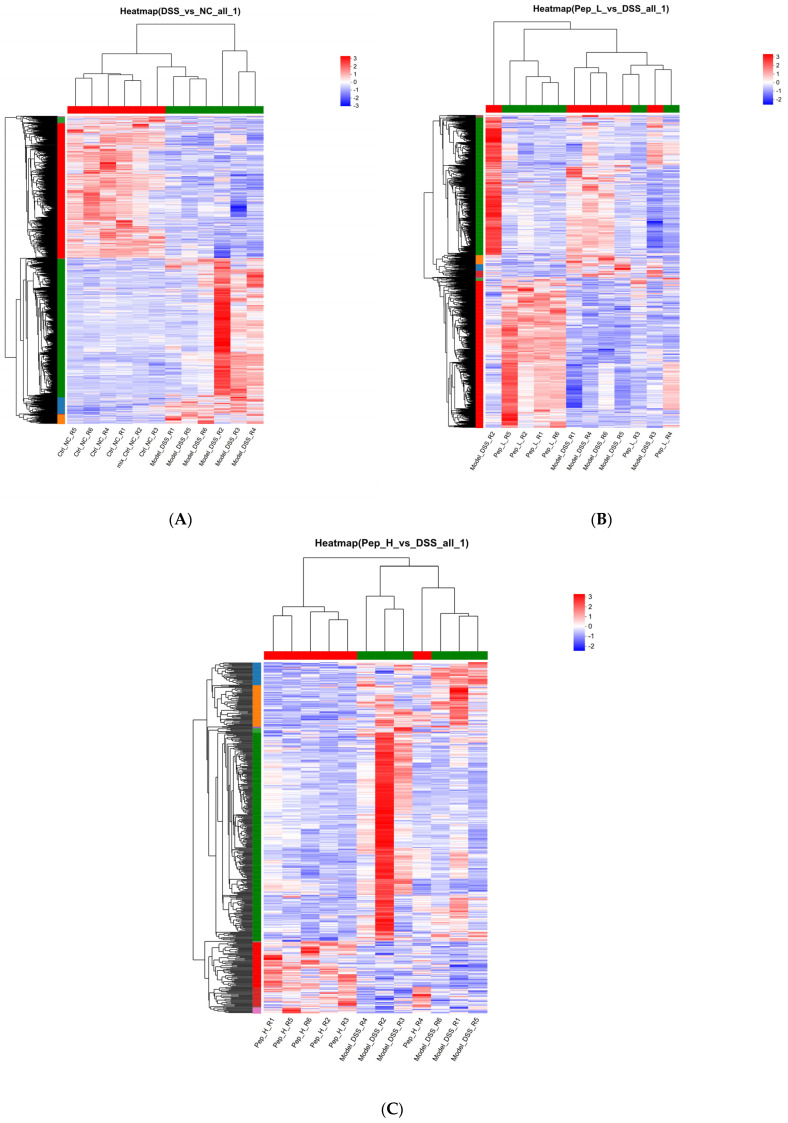
Hierarchical clustering heatmaps of differentially expressed genes. (**A**) DSS vs. NC comparison. (**B**) Pep_L vs. DSS comparison. (**C**) Pep_H vs. DSS comparison. Group abbreviations are defined in Figure 1. n = 6 per group.

The Venn diagram (Figure 12) showed 28 common DEGs among the DSS_vs_NC, Pep_L_vs_DSS, and Pep_H_vs_DSS comparisons. The Pep_L_vs_DSS comparison contained 740 specific DEGs, more than the 280 specific DEGs in the Pep_H_vs_DSS comparison, further indicating that the two AP doses differed in the extent of their effects on the colonic transcriptome. Clustering heatmaps (Figure 13A–C) showed clear separation between the DSS and NC groups. After AP intervention, the expression patterns of some genes changed, and the Pep_L and Pep_H groups displayed different clustering characteristics.

**Figure 14:**
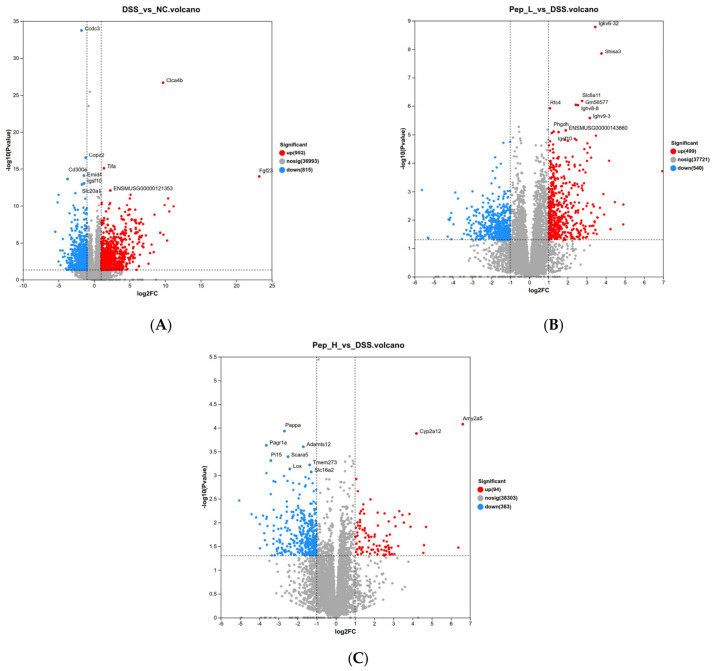
Volcano plots of differentially expressed genes among treatment comparisons. (**A**) DSS vs. NC. (**B**) Pep_L vs. DSS. (**C**) Pep_H vs. DSS. Each point represents a gene. Red and blue points indicate significantly upregulated and downregulated genes, respectively. Group abbreviations are defined in Figure 1. n = 6 per group.

Volcano plots were consistent with the DEG statistics (Figure 14A–C). The DSS_vs_NC comparison contained the largest number of DEGs, with representative genes including *Clca4b*, *Fgf23*, *Tifa*, and *Ccdc3*. In the Pep_L_vs_DSS comparison, DEGs were broadly distributed, with representative genes including *Igkv6-32*, *Shisa3*, *Slc6a11*, *Ighv8-8*, and *Ighv9-3*. The presence of *Igkv-* and *Ighv*-related genes suggests prominent immunoglobulin-related transcriptional changes. In the Pep_H_vs_DSS comparison, fewer DEGs were observed, with representative genes including *Pappa*, *Pagr1a*, *Adamts12*, *Lox*, *Scara5*, and *Slc16a2*. Genes such as *Adamts12* and *Lox* are associated with extracellular matrix remodeling, collagen maturation, and tissue structural regulation, suggesting that the high-dose group showed transcriptional features related to tissue repair processes.

#### 3.8.2. GO Enrichment Analysis: Low Dose Modulates Immunity, High Dose Affects ECM

**Figure 15:**
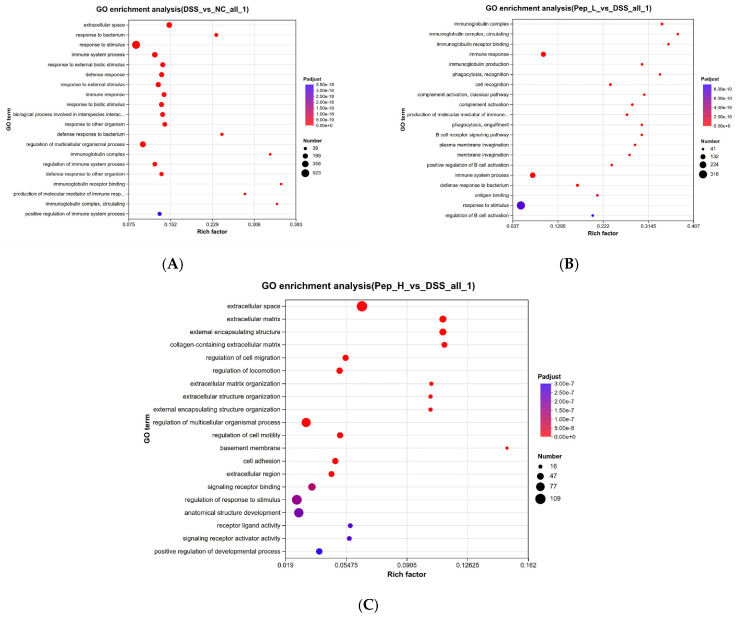
Gene Ontology enrichment analysis of differentially expressed genes. (**A**) Gene Ontology (GO) enrichment analysis of differentially expressed genes (DEGs) in the DSS vs. NC comparison. (**B**) GO enrichment analysis of DEGs in the Pep_L vs. DSS comparison. (**C**) GO enrichment analysis of DEGs in the Pep_H vs. DSS comparison. n = 6 per group.

GO enrichment analysis of DSS_vs_NC DEGs (Figure 15A) showed that the DEGs were mainly enriched in extracellular space, response to bacterium, response to stimulus, immune system process, response to external biotic stimulus, defense response, immune response, defense response to bacterium, immunoglobulin complex, and immunoglobulin receptor binding. These results indicate that bacterial response, defense response, immune system processes, and immunoglobulin-related functions were markedly altered in colon tissue after DSS treatment, consistent with the mucosal inflammation and barrier injury described above.

GO terms enriched in Pep_L_vs_DSS DEGs were mainly immune-related processes (Figure 15B), including immunoglobulin complex, circulating immunoglobulin complex, immunoglobulin receptor binding, immune response, immunoglobulin production, phagocytosis recognition, complement activation, classical pathway complement activation, B cell receptor signaling pathway, and positive regulation of B cell activation. These results indicate that the DEGs in the low-dose group were mainly associated with immunoglobulin production, B cell receptor signaling, complement activation, and phagocytic recognition. Together with the larger number of DEGs in the Pep_L group, these findings indicate that the low-dose group showed relatively broad transcriptional changes related to colonic immune responses after DSS induction.

GO terms enriched in Pep_H_vs_DSS DEGs were mainly related to extracellular matrix and tissue structure (Figure 15C), including extracellular matrix, external encapsulating structure, collagen-containing extracellular matrix, extracellular matrix organization, extracellular structure organization, basement membrane, cell adhesion, regulation of cell migration, and regulation of cell motility. These results indicate that the high-dose group showed stronger enrichment of terms related to extracellular matrix composition, basement membrane structure, cell adhesion, and cell migration. These processes are closely related to intestinal epithelial migration, tissue structural reconstruction, and mucosal repair [37,38]. In contrast to the immune-related terms enriched in the Pep_L group, these findings suggest dose-related differences in host transcriptional response patterns.

#### 3.8.3. KEGG Pathway Enrichment Analysis Showed Different Pathway Profiles Between AP-Treated Groups

**Figure 16:**
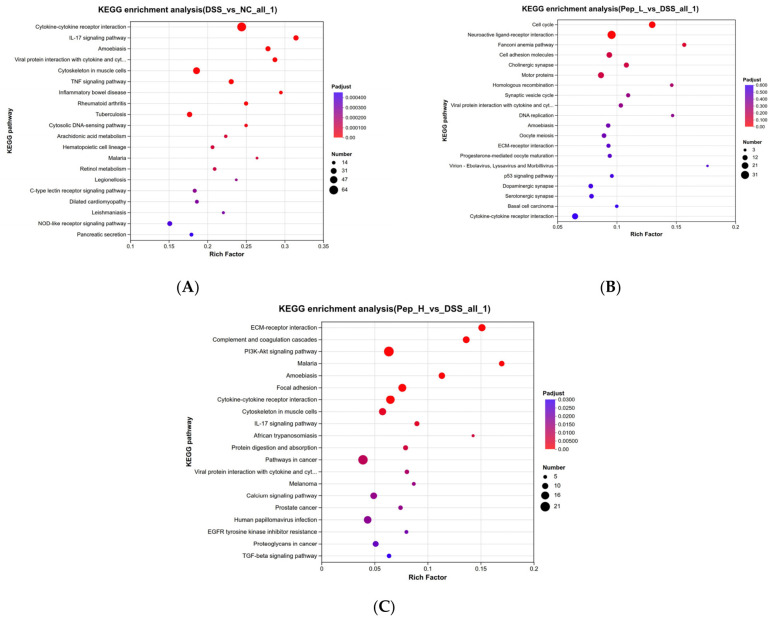
KEGG pathway enrichment analysis of differentially expressed genes. (**A**) Kyoto Encyclopedia of Genes and Genomes (KEGG) pathway enrichment analysis of differentially expressed genes (DEGs) in the DSS vs. NC comparison. (**B**) KEGG pathway enrichment analysis of DEGs in the Pep_L vs. DSS comparison. (**C**) KEGG pathway enrichment analysis of DEGs in the Pep_H vs. DSS comparison. n = 6 per group.

KEGG enrichment analysis (Figure 16A) showed that DSS_vs_NC DEGs were mainly enriched in Cytokine-cytokine receptor interaction, IL-17 signaling pathway, TNF signaling pathway, Inflammatory bowel disease, NOD-like receptor signaling pathway, C-type lectin receptor signaling pathway, Arachidonic acid metabolism, and Cytosolic DNA-sensing pathway. These pathways are closely associated with inflammatory cytokine signaling, innate immune recognition, and inflammatory mediator metabolism, indicating that DSS strongly activated inflammation-related signaling in colon tissue. Among them, TNF and IL-17 signaling pathways are important pro-inflammatory pathways in IBD pathogenesis [1].

As shown in Figure 16B, KEGG enrichment of Pep_L_vs_DSS DEGs involved Cell cycle, Neuroactive ligand-receptor interaction, Fanconi anemia pathway, Cell adhesion molecules, Homologous recombination, DNA replication, p53 signaling pathway, ECM-receptor interaction, and Cytokine-cytokine receptor interaction. Combined with the GO results, the Pep_L group was characterized by immune-related transcriptional features, together with enrichment of pathways related to cell cycle, DNA replication, and cell adhesion. These results suggest that the transcriptional changes observed in the low-dose group may be related to immune responses and cell proliferation-associated processes during recovery from DSS-induced injury.

KEGG enrichment of Pep_H_vs_DSS DEGs (Figure 16C) showed that the DEGs were mainly enriched in ECM-receptor interaction, Complement and coagulation cascades, PI3K-Akt signaling pathway, Focal adhesion, Cytokine-cytokine receptor interaction, IL-17 signaling pathway, Protein digestion and absorption, Calcium signaling pathway, and TGF-beta signaling pathway. Genes enriched in ECM-receptor interaction and Focal adhesion are related to interactions between cells and the extracellular matrix and are important for epithelial cell adhesion, migration, and tissue structural reconstruction. Genes enriched in the PI3K-Akt signaling pathway are closely associated with intestinal epithelial cell survival, proliferation, and mucosal repair. Previous studies have shown that its activation can promote intestinal epithelial regeneration and barrier recovery after inflammation [37]. The TGF-beta signaling pathway is also involved in immune regulation, epithelial repair, and matrix remodeling, although its effects depend on the inflammatory stage and cell type and require further interpretation together with the direction of specific gene expression changes.

Taken together, the GO and KEGG results indicate that both low-dose and high-dose APs were associated with changes in DSS-induced transcriptional abnormalities in colon tissue, but the enriched biological processes differed between the two AP-treated groups. The Pep_L group had more DEGs, mainly involving immunoglobulins, B cell activation, complement activation, phagocytic recognition, and some cell cycle- and DNA replication-related processes. The Pep_H group had fewer DEGs, but these DEGs were more concentrated in tissue structure-related processes, including extracellular matrix, basement membrane, cell adhesion, cell migration, genes enriched in the PI3K-Akt signaling pathway, and focal adhesion. When considered together with the microbiota results, the Pep_H group showed a distinct microbial profile and transcriptomic enrichment of pathways related to mucosal barrier repair and tissue remodeling. These findings suggest a potential association between AP-related microbiota restructuring and host repair-related transcriptional responses.

### 3.9. Correlation Analysis Among Gut Microbiota, Host Markers, and Colitis-Related Phenotypes

**Figure 17:**
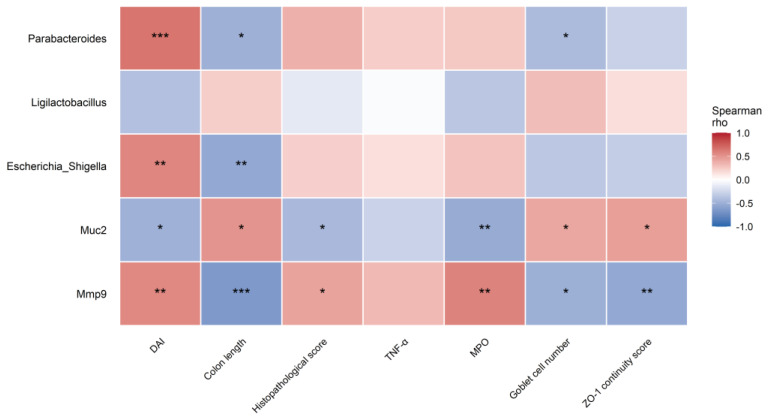
Spearman correlation analysis among selected gut microbial taxa, inflammatory markers, barrier-related indicators, and colitis phenotypes. Red indicates positive correlations and blue indicates negative correlations. Asterisks indicate statistical significance: * *p* < 0.05, ** *p* < 0.01, and *** *p* < 0.001. n = 24 biological samples, 6 mice per group.

To further examine the relationships among gut microbiota, inflammatory responses, barrier-related indicators, and disease severity, Spearman correlation analysis was performed using selected microbial taxa, representative host markers, and colitis-related phenotypic indices (Figure 17). The variables included Parabacteroides, Ligilactobacillus, Escherichia-Shigella, Muc2, and Mmp9, together with DAI, colon length, histopathological score, TNF-α, MPO, goblet cell number, and ZO-1 junctional continuity score.

The correlation matrix showed that DAI was positively correlated with histopathological score, MPO, and Mmp9, and negatively correlated with colon length, consistent with the progression of DSS-induced colonic inflammation and tissue injury. In contrast, Muc2 was positively correlated with goblet cell number and ZO-1 junctional continuity score, and negatively correlated with inflammatory and disease-severity indicators, suggesting that preservation of the mucus layer and epithelial junction integrity was associated with attenuated colitis injury.

Among the microbial taxa, Escherichia-Shigella was positively associated with disease-related indicators and negatively associated with colon length, supporting its association with the inflammatory status induced by DSS. Ligilactobacillus showed relatively weak correlations with the selected phenotypic and molecular indicators. Notably, Parabacteroides was positively correlated with DAI and negatively correlated with colon length and goblet cell number, indicating that genus-level changes in Parabacteroides should be interpreted cautiously. Overall, these results provide statistical evidence linking microbial profiles, inflammatory activity, epithelial barrier indicators, and colitis phenotypes, but the observed relationships remain correlative rather than causal.

## 4. Discussion

The present study indicates that APs attenuated DSS-induced acute colitis in mice, with effects associated with inflammatory status, oxidative stress, epithelial barrier integrity, gut microbiota structure, and host transcriptional responses. Rather than supporting a single linear mechanism, the findings suggest that APs may act through several interconnected processes. This interpretation is consistent with the current understanding that DSS-induced colitis involves epithelial injury, immune activation, oxidative stress, and disruption of host-microbiota homeostasis [1,16,18]. However, the microbiota and transcriptomic data remain associative and should be viewed as mechanistic clues rather than direct causal evidence.

The anti-inflammatory and antioxidant changes after AP intervention may be biologically linked. In DSS colitis, TNF-α and IL-6 amplify mucosal inflammation by promoting immune cell recruitment and epithelial injury, whereas IL-10 contributes to the limitation of excessive immune responses [21]. MPO activity mainly reflects neutrophil infiltration, an important source of reactive oxygen species in inflamed mucosa. Therefore, the coordinated improvement in cytokine balance, MPO activity, and SOD activity suggests that APs may reduce oxidative injury partly by limiting inflammatory cell accumulation and restoring antioxidant capacity. Similar patterns have been reported for walnut, oyster, wheat, oat, quinoa, and sea cucumber peptides in DSS-induced colitis models [5,39,40], supporting the broader view that food-derived peptides may protect the intestine through moderate regulation of multiple inflammatory, redox-related, barrier, and microbiota-associated processes [5].

Barrier preservation is likely central to the protective phenotype. The mucus layer, mainly formed by goblet cell-derived MUC2, limits direct contact between luminal microorganisms and the epithelium [26]. Tight junction proteins such as ZO-1 further maintain epithelial paracellular integrity, and ZO-1 disruption is a typical feature of DSS-induced mucosal injury [27]. Thus, the improvement in goblet cell status and ZO-1 junctional continuity suggests that APs may help restore both mucus and epithelial junction barriers, thereby reducing microbial antigen translocation and weakening the cycle of epithelial damage and mucosal inflammation. Nevertheless, ZO-1 immunohistochemistry alone is insufficient to validate the full tight junction or repair program. Future studies should examine occludin, claudins, MUC2, and epithelial repair markers at both mRNA and protein levels.

The microbiota findings should be interpreted cautiously. DSS-associated enrichment of Enterobacteriaceae-related taxa and Escherichia-Shigella is consistent with previous reports linking these bacteria to intestinal inflammation, oxidative stress, and barrier dysfunction [28,31]. AP intervention was associated with genus-level changes involving Ligilactobacillus, Escherichia-Shigella, and Parabacteroides-related taxa. Lactic acid bacteria have been reported to alleviate experimental colitis through microbiota modulation and inhibition of inflammatory signaling pathways [33]. Some studies also suggest that specific species such as Parabacteroides distasonis may regulate intestinal immunity and protect against experimental colitis [34,35]. However, the present study cannot assign such species-level functions because 16S rRNA sequencing mainly provides genus-level resolution. In addition, the correlation analysis did not support a simple protective interpretation of genus-level Parabacteroides, which was positively associated with DAI and negatively associated with colon length and goblet cell number. Therefore, Parabacteroides should be discussed as a taxonomic change rather than as a confirmed beneficial bacterium in this study.

Transcriptomic enrichment provided another layer of mechanistic clues but should not be overinterpreted. The low-dose and high-dose AP groups showed different transcriptional response patterns rather than a strict dose-dependent trend. The broader immune-related changes in the low-dose group and the stronger enrichment of extracellular matrix organization, focal adhesion, and PI3K-Akt-related pathways in the high-dose group suggest different regulatory orientations. PI3K-Akt signaling is involved in epithelial cell survival, proliferation, and regeneration, whereas ECM remodeling and focal adhesion are related to epithelial migration and mucosal reconstruction [37,38,41]. These pathways are biologically consistent with tissue repair, but enrichment analysis alone cannot prove pathway activation. Moreover, ECM remodeling may support acute repair but can contribute to fibrosis if excessive or persistent [42]. Therefore, these transcriptomic results should be described as repair-associated signatures requiring RT-qPCR and protein-level validation. Recent studies also emphasize that restoration of intestinal barrier integrity is an active therapeutic target in IBD, not merely a secondary consequence of inflammation control [43].

The added correlation analysis helps connect phenotypic, microbial, and host-response datasets, but it does not establish causality. The alignment of disease severity indicators with inflammatory markers, and the opposite pattern of barrier-related indicators, supports the internal consistency of the dataset. However, whether microbiota changes drive host transcriptional responses, whether barrier recovery reshapes the microbiota, or whether both occur in parallel after reduced injury remains unresolved. Recent DSS-colitis studies combining microbiota analysis with pathway validation, fecal microbiota transplantation, or multi-omics integration provide useful models for future work [44,45]. For APs, fecal microbiota transplantation, antibiotic depletion, gnotobiotic models, shotgun metagenomics, fecal metabolomics, and strain-level intervention would be needed to test causal microbiota–host relationships.

Several limitations should be acknowledged. First, APs were used as a complex peptide mixture, and the active sequences responsible for the in vivo effects were not identified in this manuscript. Further characterization of molecular weight distribution, peptide purity, amino acid composition, major sequences, and batch consistency is required. Second, only male BALB/c mice and a 7-day acute DSS model were used, limiting extrapolation to chronic relapsing UC, sex-related variability, and clinical settings. Third, the absence of a positive control such as mesalazine or sulfasalazine limits comparison with established treatments. Fourth, digestive stability, bioavailability, toxicological safety, acceptability, human-equivalent dose, and industrial scalability were not evaluated. These issues are essential before APs can be considered for nutritional or translational application.

In conclusion, APs alleviated DSS-induced acute colitis in mice, and this effect was associated with reduced inflammatory and oxidative stress burden, improved epithelial barrier indicators, altered gut microbiota profiles, and repair-related transcriptional signatures. These findings support almond protein as a potential source of bioactive peptides for intestinal health. At the same time, the proposed microbiota–host and pathway-related mechanisms remain hypotheses requiring peptide characterization, targeted molecular validation, and causal microbiota experiments.

## Figures and Tables

**Table 1:** Disease activity index scoring criteria.

Body Weight Loss	Stool Consistency	Rectal Bleeding	Score
<1%	Normal	Normal	0
1–5%	Soft but formed	Occult blood positive	1
5–10%	Soft	Slight bleeding	2
10–15%	Mild diarrhea	Bleeding	3
>15%	Watery diarrhea	Gross bleeding	4

## Data Availability

The original data presented in the study are openly available in NCBI under the accession numbers PRJNA1472308 and PRJNA1472542.

## Abbreviations

The following abbreviations are used in this manuscript:

DSS	Dextran sulfate sodium
NC	Normal control group
DSS group	Dextran sulfate sodium-induced model group
Pep_L	Low-dose almond-derived peptide group
Pep_H	High-dose almond-derived peptide group
16SrRNA	16S ribosomal RNA
RNA-seq	RNA sequencing
DEGs	Differentially expressed genes
ASVs	Amplicon sequence variants
PCoA	Principal coordinate analysis
GO	Gene Ontology
KEGG	Kyoto Encyclopedia of Genes and Genomes
NCBI	National Center for Biotechnology Information
SRA	Sequence Read Archive

## References

[1] Neurath M.F. (2014). Cytokines in inflammatory bowel disease. Nat. Rev. Immunol..

[2] Ng S.C., Shi H.Y., Hamidi N., Underwood F.E., Tang W., Benchimol E.I., Panaccione R., Ghosh S., Wu J.C.Y., Chan F.K.L. (2017). Worldwide incidence and prevalence of inflammatory bowel disease in the 21st century: A systematic review of population-based studies. Lancet.

[3] Roda G., Ng S.C., Kotze P.G., Argollo M., Panaccione R., Spinelli A., Kaser A., Peyrin-Biroulet L., Danese S. (2020). Crohn’s disease. Nat. Rev. Dis. Prim..

[4] Rivera-Jimenez J., Berraquero-Garcia C., Perez-Galvez R., Garcia-Moreno P.J., Espejo-Carpio F.J., Guadix A., Guadix E.M. (2022). Peptides and protein hydrolysates exhibiting anti-inflammatory activity: Sources, structural features and modulation mechanisms. Food Funct..

[5] Lv R.Z., Sun N., Mao C.W., Zheng Z.H., Lin S.Y. (2024). Prevention and potential repair of colitis: Beneficial effects and regulatory mechanisms of food-derived anti-inflammatory peptides. Crit. Rev. Food Sci. Nutr..

[6] Mishima M.D.V., Martino H.S.D., Meneguelli T.S., Tako E. (2024). Effect of food derived bioactive peptides on gut health and inflammatory mediators in vivo: A systematic review. Crit. Rev. Food Sci. Nutr..

[7] Guo H.X., Ji Z.H., Wang B.B., Ren J.W., Gao W., Yuan B. (2024). Walnut peptide ameliorates DSS-induced colitis in mice by inhibiting inflammation and modulating gut microbiota. J. Funct. Foods.

[8] Qu C.F., Liang C., Liu T.L., He Y.Y., Ke K., Miao J.L. (2024). Natural zinc-rich oyster peptides ameliorate DSS-induced colitis via antioxidation, anti-inflammation, intestinal barrier repair, microbiota modulation, and SCFA accumulation. Food Biosci..

[9] Xie W.Y., Ji Z.H., Ren W.Z., Zhao P.S., Wei F.H., Hu J.P., Yuan B., Gao W. (2024). Wheat peptide alleviates DSS-induced colitis by activating the Keap1-Nrf2 signaling pathway and maintaining the integrity of the gut barrier. Food Funct..

[10] Khan A.I., Rehman A.U., Farooqui N.A., Siddiqui N.Z., Ayub Q., Ramzan M.N., Wang L., Xin Y. (2022). Effects of Shrimp Peptide Hydrolysate on Intestinal Microbiota Restoration and Immune Modulation in Cyclophosphamide-Treated Mice. Molecules.

[11] Garcia-Perez P., Xiao J.B., Munekata P.E.S., Lorenzo J.M., Barba F.J., Rajoka M.S.R., Barros L., Sprea R.M., Amaral J.S., Prieto M.A. (2021). Revalorization of Almond By-Products for the Design of Novel Functional Foods: An Updated Review. Foods.

[12] Barreca D., Nabavi S.M., Sureda A., Rasekhian M., Raciti R., Silva A.S., Annunziata G., Arnone A., Tenore G.C., Süntar I. (2020). Almonds (Prunus Dulcis Mill. D. A. Webb): A Source of Nutrients and Health-Promoting Compounds. Nutrients.

[13] Udenigwe C.C., Je J.Y., Cho Y.S., Yada R.Y. (2013). Almond protein hydrolysate fraction modulates the expression of proinflammatory cytokines and enzymes in activated macrophages. Food Funct..

[14] Liu R.L., Ge X.L., Gao X.Y., Zhan H.Y., Shi T., Su N., Zhang Z.Q. (2016). Two angiotensin-converting enzyme-inhibitory peptides from almond protein and the protective action on vascular endothelial function. Food Funct..

[15] Mirzapour M., Rezaei K., Sentandreu M.A. (2017). Identification of Potent ACE Inhibitory Peptides from Wild Almond Proteins. J. Food Sci..

[16] Park M.Y., Ji G.E., Sung M.K. (2012). Dietary Kaempferol Suppresses Inflammation of Dextran Sulfate Sodium-Induced Colitis in Mice. Dig. Dis. Sci..

[17] Kim J.J., Shajib M.S., Manocha M.M., Khan W.I. (2012). Investigating Intestinal Inflammation in DSS-induced Model of IBD. J. Vis. Exp..

[18] Perse M., Cerar A. (2012). Dextran Sodium Sulphate Colitis Mouse Model: Traps and Tricks. J. Biomed. Biotechnol..

[19] Guo H.X., Xie W.Y., Ji Z.H., Wang B.B., Ren W.Z., Gao W., Yuan B. (2024). Oyster Peptides Ameliorate Dextran Sulfate Sodium-Induced Ulcerative Colitis via Modulating the Gut Microbiota and Inhibiting the TLR4/NF-κB Pathway. Nutrients.

[20] Qiu W.P., Wang Z.C., Liu Q.R., Du Q.W., Zeng X.Q., Wu Z., Pan D.D., Zhang X.H., Tu M.L. (2024). Structure and regulatory mechanisms of food-derived peptides in inflammatory bowel disease: A review. Food Sci. Nutr..

[21] Kuhn R., Lohler J., Rennick D., Rajewsky K., Muller W. (1993). Interleukin-10-deficient mice develop chronic enterocolitis. Cell.

[22] Papadakis K.A., Krempski J., Svingen P., Xiong Y.N., Sarmento O.F., Lomberk G.A., Urrutia R.A., Faubion W.A. (2015). Kruppel-like factor KLF10 deficiency predisposes to colitis through colonic macrophage dysregulation. Am. J. Physiol.-Gastrointest. Liver Physiol..

[23] Spittau B., Krieglstein K. (2012). Klf10 and Klf11 as mediators of TGF-beta superfamily signaling. Cell Tissue Res..

[24] Zou T.B., He T.P., Li H.B., Tang H.W., Xia E.Q. (2016). The Structure-Activity Relationship of the Antioxidant Peptides from Natural Proteins. Molecules.

[25] Ji Z.H., Xie W.Y., Zhao P.S., Wu H.Y., Ren W.Z., Hu J.P., Gao W., Yuan B., Cacciola N.A., Borrelli F. (2023). Oat Peptides Alleviate Dextran Sulfate Sodium Salt-Induced Colitis by Maintaining the Intestinal Barrier and Modulating the Keap1-Nrf2 Axis. Nutrients.

[26] Van der Sluis M., De Koning B.A.E., De Bruijn A., Velcich A., Meijerink J.P.P., Van Goudoever J.B., Büller H.A., Dekker J., Van Seuningen I., Renes I.B. (2006). Muc2-deficient mice spontaneously develop colitis, indicating that Muc2 is critical for colonic protection. Gastroenterology.

[27] Poritz L.S., Garver K.I., Green C., Fitzpatrick L., Ruggiero F., Koltun W.A. (2007). Loss of the tight junction protein ZO-1 in dextran sulfate sodium induced colitis. J. Surg. Res..

[28] Munyaka P.M., Rabbi M.F., Khafipour E., Ghia J.E. (2016). Acute dextran sulfate sodium (DSS)-induced colitis promotes gut microbial dysbiosis in mice. J. Basic Microbiol..

[29] Rooks M.G., Veiga P., Wardwell-Scott L.H., Tickle T., Segata N., Michaud M., Gallini C.A., Beal C., van Hylckama-Vlieg J.E.T., Ballal S.A. (2014). Gut microbiome composition and function in experimental colitis during active disease and treatment-induced remission. ISME J..

[30] Sommer F., Anderson J.M., Bharti R., Raes J., Rosenstiel P. (2017). The resilience of the intestinal microbiota influences health and disease. Nat. Rev. Microbiol..

[31] Baldelli V., Scaldaferri F., Putignani L., Del Chierico F. (2021). The Role of Enterobacteriaceae in Gut Microbiota Dysbiosis in Inflammatory Bowel Diseases. Microorganisms.

[32] Lebeer S., Vanderleyden J., De Keersmaecker S.C.J. (2008). Genes and Molecules of Lactobacilli Supporting Probiotic Action. Microbiol. Mol. Biol. Rev..

[33] Xia Y.J., Chen Y., Wang G.Q., Yang Y.J., Song X., Xiong Z.Q., Zhang H., Lai P., Wang S.J., Ai L.Z. (2020). Lactobacillus plantarum AR113 alleviates DSS-induced colitis by regulating the TLR4/MyD88/NF-κB pathway and gut microbiota composition. J. Funct. Foods.

[34] Kverka M., Zakostelska Z., Klimesova K., Sokol D., Hudcovic T., Hrncir T., Rossmann P., Mrazek J., Kopecny J., Verdu E.F. (2011). Oral administration of Parabacteroides distasonis antigens attenuates experimental murine colitis through modulation of immunity and microbiota composition. Clin. Exp. Immunol..

[35] Gaifem J., Mendes-Frias A., Wolter M., Steimle A., Garzón M.J., Ubeda C., Nobre C., González A., Pinho S.S., Cunha C. (2024). Akkermansia muciniphila and Parabacteroides distasonis synergistically protect from colitis by promoting ILC3 in the gut. Mbio.

[36] Shaw L.P., Bassam H., Barnes C.P., Walker A.S., Klein N., Balloux F. (2019). Modelling microbiome recovery after antibiotics using a stability landscape framework. ISME J..

[37] Ma Y.J., Lang X.M., Yang Q., Han Y., Kang X., Long R., Du J.X., Zhao M.M., Liu L.H., Li P.T. (2023). Paeoniflorin promotes intestinal stem cell-mediated epithelial regeneration and repair via PI3K-AKT-mTOR signalling in ulcerative colitis. Int. Immunopharmacol..

[38] Mortensen J.H., Lindholm M., Langholm L.L., Kjeldsen J., Bay-Jensen A.C., Karsdal M.A., Manon-Jensen T. (2019). The intestinal tissue homeostasis: The role of extracellular matrix remodeling in inflammatory bowel disease. Expert Rev. Gastroenterol. Hepatol..

[39] Wang L.F., Wu S.F., Chen T., Xiong L., Wang F., Song H.Z., Zhou J.X., Wei S.X., Ren B., Shen X.C. (2024). A quinoa peptide protects impaired mucus barriers in colitis mice by inhibiting NF-κB-TRPV1 signaling and regulating the gut microbiota. Food Funct..

[40] Yu S., Guo H.X., Ji Z.H., Zheng Y., Wang B.B., Chen Q.Q., Tang H.Y., Yuan B. (2023). Sea Cucumber Peptides Ameliorate DSS-Induced Ulcerative Colitis: The Role of the Gut Microbiota, the Intestinal Barrier, and Macrophage Polarization. Nutrients.

[41] Biancheri P., Di Sabatino A., Corazza G.R., MacDonald T.T. (2013). Proteases and the gut barrier. Cell Tissue Res..

[42] Rieder F., Fiocchi C. (2009). Intestinal fibrosis in IBD-a dynamic, multifactorial process. Nat. Rev. Gastroenterol. Hepatol..

[43] Kong C., Yang M.F., Yue N.N., Zhang Y., Tian C.M., Wei D.R., Shi R.Y., Yao J., Wang L.S., Li D.F. (2024). Restore Intestinal Barrier Integrity: An Approach for Inflammatory Bowel Disease Therapy. J. Inflamm. Res..

[44] Chen J.H., Zhao C.L., Li Y.S., Yang Y.B., Luo J.G., Zhang C., Wang L. (2023). Moutai Distiller’s grains Polyphenol extracts and rutin alleviate DSS-induced colitis in mice: Modulation of gut microbiota and intestinal barrier function (R2). Heliyon.

[45] Hong H.H., Lee S.Y., Jang D.H., Ju S.E., Shim J.E., Kim T.H., Kang H.S., Kim S.M. (2025). Multiomics Integration Reveals Microbial Gene Interactions Shaping Host Responses in a DSS-Induced Colitis Mouse Model. J. Microbiol. Biotechnol..

